# Employing Computers for the Recruitment into Clinical Trials: A Comprehensive Systematic Review

**DOI:** 10.2196/jmir.3446

**Published:** 2014-07-01

**Authors:** Felix Köpcke, Hans-Ulrich Prokosch

**Affiliations:** ^1^Center for Information and CommunicationUniversity Hospital ErlangenErlangenGermany; ^2^Department for Medical InformaticsFriedrich-Alexander University Erlangen-NurembergErlangenGermany

**Keywords:** automation, clinical trials as topic, decision support systems, clinical, patient selection, research subject recruitment

## Abstract

**Background:**

Medical progress depends on the evaluation of new diagnostic and therapeutic interventions within clinical trials. Clinical trial recruitment support systems (CTRSS) aim to improve the recruitment process in terms of effectiveness and efficiency.

**Objective:**

The goals were to (1) create an overview of all CTRSS reported until the end of 2013, (2) find and describe similarities in design, (3) theorize on the reasons for different approaches, and (4) examine whether projects were able to illustrate the impact of CTRSS.

**Methods:**

We searched PubMed titles, abstracts, and keywords for terms related to CTRSS research. Query results were classified according to clinical context, workflow integration, knowledge and data sources, reasoning algorithm, and outcome.

**Results:**

A total of 101 papers on 79 different systems were found. Most lacked details in one or more categories. There were 3 different CTRSS that dominated: (1) systems for the retrospective identification of trial participants based on existing clinical data, typically through Structured Query Language (SQL) queries on relational databases, (2) systems that monitored the appearance of a key event of an existing health information technology component in which the occurrence of the event caused a comprehensive eligibility test for a patient or was directly communicated to the researcher, and (3) independent systems that required a user to enter patient data into an interface to trigger an eligibility assessment. Although the treating physician was required to act for the patient in older systems, it is now becoming increasingly popular to offer this possibility directly to the patient.

**Conclusions:**

Many CTRSS are designed to fit the existing infrastructure of a clinical care provider or the particularities of a trial. We conclude that the success of a CTRSS depends more on its successful workflow integration than on sophisticated reasoning and data processing algorithms. Furthermore, some of the most recent literature suggest that an increase in recruited patients and improvements in recruitment efficiency can be expected, although the former will depend on the error rate of the recruitment process being replaced. Finally, to increase the quality of future CTRSS reports, we propose a checklist of items that should be included.

## Introduction

 Medical progress depends on the evaluation of new diagnostic and therapeutic interventions within clinical trials. The value of each clinical trial depends on the successful recruitment of patients within a limited time frame. The number of participants must be sufficiently large to allow for scientifically and statistically valid analysis. Unfortunately, many trials experience gaps between initially planned and finally achieved participant numbers or they need to prolong their recruitment period. Slow recruitment delays medical progress and leads to unnecessarily high study costs [1-3].

The main stakeholders in the recruitment process are the patient, the treating physician, the study nurse, and the principal investigator. But when it comes to the details of how responsibilities and tasks are distributed and how stakeholders interact with one another, recruitment processes start to show large variability. These specifics are influenced by a multitude of factors, including whether the trial is prospective or retrospective, the number of patients to be screened, the fraction of potential participants among the screened patients, the number of participating clinics, the urgency of recruiting a patient after discovering eligibility, the local data protection laws, the available funds or the organization, and infrastructure of the clinical institutions which pursue the trial.

Because of this variability in the recruitment processes, numerous reasons for failure to include sufficient participants into a trial were found [4-6]. On the most abstract level, these are overoptimistic feasibility estimations of future eligible patient numbers [7,8], the inability to motivate physicians to approach their patients [9-12], and the inability to motivate patients to participate [13,14].

Following increased levels of patient data capture in digital systems and the advent of clinical decision support systems, the early 1990s also saw the use of computers for matching patients and trial protocols. These clinical trial recruitment support systems (CTRSS) aim to solve the issue of false feasibility estimations, to generate a positive impact on the treating physicians’ enrollment efforts, and to reduce the resources required to set up a successful recruitment process. Although many CTRSS have been proposed, the problems in recruitment persist [15,16].

In this context, Cuggia et al [17] raised the question “What significant work has been carried out toward automating patient recruitment?” and reviewed the literature published between 1998 and October 2009. They found a comparatively small number of papers related to 28 distinct CTRSS. Most of these projects had focused on the technological feasibility of the search algorithm and neglected assessments of the system’s impact on recruitment in real-life scenarios. Cuggia et al concluded “that the automatic recruitment issue is still open” and that in 2009 it was still “difficult to make any strong statements about how effective automatic recruitment is, or about what makes a good decision support system for clinical trial recruitment.”

Since then, CTRSS have become even more popular. Many independent institutions have tackled the challenge to improve their local recruitment processes. Large European collaborations, such as Electronic Health Records for Clinical Research (EHR4CR) [18], and national collaborations, for example in Germany [19], have been initiated to create information technology (IT)-supported patient recruitment architectures and platforms. For the related but broader challenge of extracting meaningful patient information from electronic health record (EHR) data, a plethora of publications have been published in recent years and the term *patient phenotyping* has been coined [20]. Recently, Shivade et al [21] presented a review on phenotyping techniques. They observed “a rise in the number of studies associated with cohort identification using electronic medical records.”

The rapidly growing knowledge about and the importance of electronic patient recruitment systems warrants a new review of the existing literature. Our objectives were to (1) create an overview of all papers published until the end of 2013, (2) find and describe similarities in CTRSS design, (3) discuss the reasons for different approaches, and (4) examine whether new projects were able to illustrate the impact of CTRSS.

## Methods

### Search Strategy

One of the authors (FK) searched the database PubMed with 2 queries. The first query contained keywords for publication titles and Medical Subject Headings (MeSH) terms. Because most recent articles were not yet completely indexed with MeSH terms, a second query performed a more profound keyword search in all fields. Neither query was limited to a specific time period:

1. PubMed query 1: (“clinical trial”[Title] OR “clinical trials”[Title] OR “Clinical Trials as Topic”[MESH]) AND (“eligibility”[Title] OR “identification”[Title] OR “recruitment”[Title] OR “Patient Selection”[MESH] OR “cohort”[Title] OR “accrual”[Title] OR “enrollment”[Title] OR “enrolment” [Title] OR “screening”[Title]) AND (“electronic”[Title] OR “computer”[Title] OR “software”[Title] OR “Decision Making, Computer-Assisted”[MESH] OR “Decision Support Systems, Clinical”[Mesh] OR “Medical Records Systems, Computerized”[Mesh])
2. PubMed query 2: (“clinical trial”[All Fields] OR “clinical trials”[All Fields]) AND (“eligibility”[All Fields] OR “identification”[All Fields] OR “recruitment”[All Fields] OR “accrual”[All Fields] OR “enrollment”[All Fields] OR “enrolment”[All Fields] OR “screening”[All fields]) AND (“participants”[All Fields] OR “cohort”[All fields] OR “patients”[All Fields]) AND (“electronic”[All fields] OR “computer”[All fields] OR “software”[All fields] OR “automatic”[All Fields])

Both queries were executed on January 15, 2014. After removing all duplicates from the combined result sets of both queries, FK screened titles and abstracts for the inclusion criteria. We then tried to obtain the full text of all included articles for a second screening. Finally, FK reviewed all references of the included manuscripts for additional articles. In case of uncertainty about the inclusion of an article, it was discussed with HUP for a final decision.

### Inclusion Criteria

Our review covers primary research articles and conference proceedings on computer systems that compared patient data and eligibility criteria of a clinical trial to identify either potential participants for a given trial or suitable trials for a given patient. The system must have employed a computer to determine patient eligibility; that is, the utilization of electronically captured data was insufficient if the matchmaking process itself was done manually (eg, [22,23]). Manual processes before and after eligibility determination were otherwise accepted. Articles on the construction and processing of eligibility criteria, although closely tied to the construction and usage of CTRSS, were not part of this review (eg, [24,25]). Although technically the same, we also excluded decision support systems that identified patients for other purposes than clinical trials recruitment (eg, for diagnosing [26] or phenotyping [27]).

### Classification

The classification of CTRSS was roughly based on that of a previous review by Cuggia et al [17] to render results comparable with one another. They included (1) the clinical context or setting to which the system was deployed, (2) the manner of integration into the existing clinical or recruitment workflow, (3) the source and format of patient data and eligibility criteria, (4) the reasoning method employed to derive eligible patients, and (5) the outcome obtained by the system’s application to one or more clinical trials.

## Results

### Included Studies

**Figure 1:**
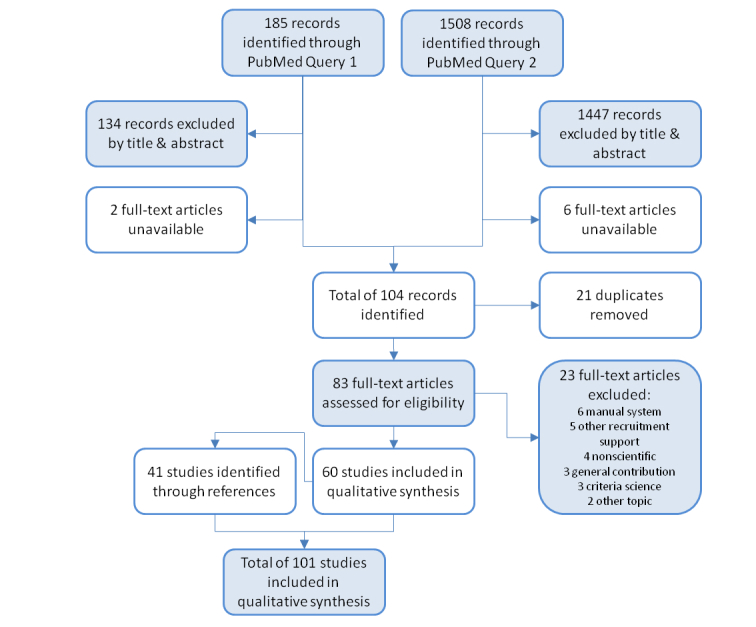
Flow diagram on the process of literature selection.

The 2 PubMed queries together yielded 1693 articles. A total of 1581 articles were removed from the literature pool based on their titles and abstracts. After removal of 8 articles that could not be obtained as full text and 21 duplicates, we arrived at 83 distinct articles, of which 60 were included in the qualitative analysis after review. In all, 5 of the excluded articles described other supportive measures for trial recruitment, 4 were deemed nonscientific (eg, commentaries), 6 described manual systems or the mode of eligibility determination was not clearly stated, 3 constituted general contributions without a relation to a specific CTRSS, 3 focused on the representation of eligibility criteria in a computable format, and 2 articles dealt with other topics (eg, phenotyping, personalized medicine). We obtained 41 additional articles through references and arrived at a final pool of 101 articles [3,28-127] on 79 different systems. The Preferred Reporting Items for Systematic Reviews and Meta-Analyses (PRISMA) flow diagram [128] in Figure 1 shows the different phases of the article selection process.

### Results Structure

Multimedia Appendix 1 shows a list of all articles grouped by system and ordered by first publication date (objective 1). It also summarizes the CTRSS characteristics according to the categories described subsequently. In the following sections on CTRSS characteristics, we identify and describe CTRSS groups with similar features (objective 2). We also speculate on environmental characteristics that led the developers to favor a group or reject another (objective 3). All evidence for the impact of CTRSS on patient recruitment is presented in Outcomes (objective 4).

### Characteristics of Included Articles

**Figure 2:**
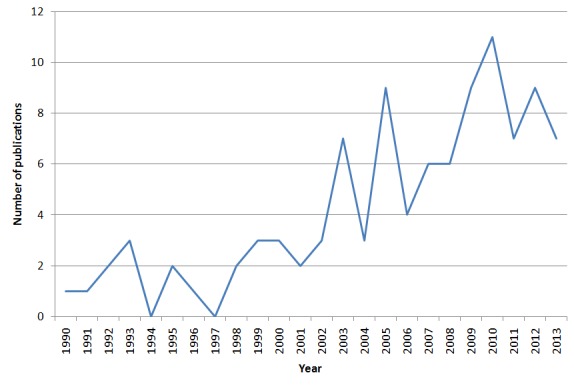
Number of publications on CTRSS per year.

Regarding system maturity, 12 articles reported on a CTRSS concept that was not implemented yet. A total of 42 articles described a prototypical implementation, often including performance tests, but no application to a running clinical trial. Another 47 articles described fully matured systems that were used to recruit patients into at least 1 trial. First publications on CTRSS dated back to 1990. However, there were no more than 3 publications per year until 2003. Since then, 7 articles per year were published on average, so that nearly 80% of all articles were from the past 10 years (Figure 2).

### Clinical Context and Scope of Application

CTRSS have been implemented and used in trials in a wide variety of clinical domains. Still, many systems were evaluated for only 1 trial or trials from the same domain. With 17 CTRSS in this domain, oncology (especially breast cancer) was found particularly often. This domain may be favorable because it is research intensive with many open trials and exceptionally large available volumes of patient data and funding. The functionalities and algorithms of the CTRSS seemed largely independent from the clinical domain. Thus, no author precluded the use of their system for clinical trials from other domains and many actually suggested it.

The accuracy of a CTRSS depends on the available patient data and its effect depends on the organizational environment in which it operates. Therefore, each CTRSS should be evaluated for a large number of trials and at multiple sites to increase the reliability of reported results if possible. Many authors observed this: 43 articles reported on using their system for more than 1 trial (11 did not name an exact figure) and 14 CTRSS were intended for use at multiple sites. In comparison, 37 reports evaluated a CTRSS for a single trial and 62 CTRSS were used at a single institution. In all, 11 papers failed to give the number of trials their CTRSS had been evaluated or used for.

### Workflow Integration

#### Overview

Every CTRSS has 2 points of contact with the recruitment workflow of a clinical trial. The first is the trigger that causes the system to assess the eligibility of one or more patients. The second is the communication of the assessment’s results (eg, a list of potential trial participants) to the system’s user.

#### Trigger

One way to trigger the eligibility assessment is to have the user or an administrator execute a manual process. Manual triggers are both the easiest to implement and the most commonly found. They are sufficient for cases in which patient data are entered into the CTRSS by the user who can subsequently view patient eligibility in an interactive fashion. The user can be a physician [38,88] or the patient [59,79,112]. Manual triggers are also sufficient for cases in which an eligibility assessment is required only once to generate a patient list, which is not expected to change during the trial’s recruitment phase. The latter is generally the case for retrospective trials and feasibility studies. Typical examples include Payne et al [97], Thadani et al [115], and Köpcke et al [69] who required an administrator to develop a Structured Query Language (SQL)-based query. Based on 16 years of COSTAR research queries, Murphy [85] created the graphical interface Informatics for Integrating Biology and the Bedside (i2b2) to allow investigators to parameterize query templates themselves.

For trials that require regular re-evaluation of patient eligibility because of changing patient data over time, manual triggers are generally inefficient and are replaced by automatic triggers. Automatic triggers can start eligibility assessments periodically at given time intervals [40,122] or in reaction to particular events in the hospital information system (HIS) [28,44,48,60]. Time-based triggers are generally easier to implement than event-based triggers. The interval length between assessments depends on the requirements of each trial and the computing time required for an assessment. It is usually set to a value between several minutes and 1 day. For trials that require an immediate reaction to new patient data by trial staff and for trials with comparatively rare potential participants, trigger events are preferred. Such triggers include the availability of new data or the admission of a patient.

#### Communication

The results of an eligibility assessment must be communicated somehow to the CTRSS user. The primary factor of influence when choosing a mode of communication is the target user group. If patients are supposed to use the CTRSS, it is most common to offer a separate user interface that interactively displays potentially fitting trials and/or a score indicating the patient’s fit with a certain trial [32,59,72,79,109,112,125]. Exceptions are found if the patient is interested in future trials instead of ones that are currently recruiting. In these cases, patients enter their health data into a registry or a personal health record and they are notified by email as soon as a fitting trial is detected [61,125]. If the CTRSS has no clinical/research user (ie, the direct user is IT staff), it usually transforms the raw result of the reasoning algorithm into a patient list which is subsequently handed out to the researcher [60,80,103,105,115,119]. This is the preferred mode of communication if eligibility assessments are only required once [69,97]. However, when the target users are either treating physicians or clinical investigators, the mode of communication also needs to accommodate data security regulations and the trial’s temporal requirements. Pagers seem to be the only option if the user needs to react immediately to new patient data, such as critical laboratory values [41,63]. When time is of less importance, emails are chosen to deliver both proposals for single patients and patient sets alike [34,45,106,124]. A recurring scenario is that the physician or nurse is reminded of a trial during their first consultation. To achieve this, alerts or flags are placed in the EHR which appear at a convenient moment and often allow direct evaluation of the patient’s eligibility [104,121].

When coupled with simultaneous messages, automatic triggers have the disadvantage of easily initiating alerts or prompts at a time when the user is not prepared to answer them. Untimely messages will cause the receiver to ignore them. The same effect occurs for systems with a large share of false positive alerts. This alert fatigue is regularly mentioned as a problem for CTRSS efficiency and acceptance. Numbers for the fraction of alerts that are actually reviewed by the receiver range from 25% [60], more than 30% to 40% [52], and 56% [105] to less than 70% [62]. For Ruffin [105], even “numerous prompts and reminders and customized requests” could not solve the problem. Additionally, Embi and Leonard [52] found that response rates declined at a rate of 2.7% per 2-week time period.

### Knowledge Representation and Data Sources

#### Overview

The core technical functionality of a CTRSS is the comparison of eligibility criteria with the electronically available patient data. According to Weng et al [124], the process is characterized by 3 aspects: “the expression language for representing eligibility rules, the encoding of eligibility concepts, and the modeling of patient data.” The underlying problem is that eligibility criteria are almost always given in narrative form and need to be translated into a structure that can be interpreted by the CTRSS. The same is true for the patient data, which needs to be analyzed to identify concepts that match the eligibility concepts before developing the eligibility rules themselves.

#### Source of Patient Data

Most authors choose the data source for their CTRSS according to availability and accessibility. Few CTRSS designs are based on a comparison of different potential data sources (eg, for timeliness or comprehensiveness). Nevertheless, the reuse of existing patient data for the purpose of recruitment is common practice: 64 CTRSS relied on data that was collected for other purposes originally. A total of 5 monitored the health level 7 (HL7) messages of a clinical information system, 46 of them read patient data directly from the EHR of the hospital or general practitioner, 12 used a data warehouse, and 1 used a clinical registry. In this order, these data sources increasingly collect and integrate patient data over time, software applications, and institutions, which makes access to the data of large patient sets comparatively easy. However, more integrated data often means the data source becomes increasingly detached from its origin as well (ie, some information is lost during processing and delays between the documented event and availability of the corresponding data grow). For some trials, such delays are unacceptable because trial staff need to be notified as soon as possible for specific events. Specialty subsystems, such as an electronic tracking board [3,29] or the messages exchanged between these systems [54,76,98,101,121], need to be monitored directly in these cases. A total of 3 CTRSS preloaded patient characteristics from the EHR and prompted the physician to complete missing data [90,94,95]. Wilcox et al [125] conceptualized a CTRSS that integrated EHR data and the personal health record of a patient. Only 16 CTRSS made exclusive use of data that were entered directly into the system itself by the physician (n=8), the patient (n=7), or an investigator (n=1).

#### Terminologies

The CTRSS developer can choose the terminology for clinical concept names arbitrarily if patient data are entered only for the purpose of eligibility assessment. However, if patient data are taken from an already existing data source, most developers chose to reuse the terminologies found there. A total of 66 articles did not mention the use of any terminology. Of these, 5 performed pure free-text analysis and did not necessarily require terminologies. Of those papers that did mention the use of a specific terminology, 16 named the International Classification of Diseases (ICD). This makes sense because it is also the terminology most commonly used within EHRs. There were no other widespread terminologies used for CTRSS. The Unified Medical Language System (UMLS) appeared in 6 publications and the Medical Entities Dictionary (MED), Systematized Nomenclature of Medicine Clinical Terms (SNOMED CT), and Logical Observation Identifiers Names and Codes (LOINC) in only 3, respectively. In all, 10 terminologies were each used in only 1 CTRSS, such as Cerner Multum, National Drug Code, Hospital International Classification of Diseases Adapted, Read, NCI-Thesaurus (NCI-T), and 837 billing data, or in 2 CTRSS, such as MeSH, NCI Common Data Elements (CDE), and Current Procedural Terminology.

#### Intermediary Criteria Format

Terminologies are usually chosen to suit the available patient data, whereas the intermediary criteria format is strongly associated with the reasoning method of the CTRSS. The SQL is the most frequently found representation of criteria logic. Unfortunately, the CTRSS literature lacks details on the representation of criteria expressions. A comparison of the eligibility criteria as given in the study protocol and their representation in the CTRSS is rare; 49 papers gave no information on the chosen format of eligibility expressions.

#### Translation Process

With a few exceptions, the translation process to make eligibility criteria processable for the computer seemed to be a manual one. For 51 CTRSS, the administrator was responsible for reading the trial protocol, mapping clinical concepts to the target terminology, and creating eligibility expressions. This is the most efficient process in clinical settings that generate few trials per researcher because teaching costs are minimized and experience is concentrated in 1 person. Yet, a notable fraction of the CTRSS offered the user an interface to select eligibility criteria autonomously from a small [38] or large [43] set of predefined criteria. Having the user translate the eligibility criteria of a trial is primarily meaningful for feasibility studies, giving a researcher the means to dynamically modify the criteria for a new trial and to instantly receive feedback for the change’s influence on the expected number of participants.

Lonsdale et al [75] proposed natural language processing (NLP) to support the translation process. They read eligibility sentences from the trial registry ClinicalTrials.gov, parsed them to retrieve logical forms and mapped concepts to standard terminologies to generate executable Arden syntax Medical Logic Modules (MLMs). The process succeeded for 16% of all criteria from 85 randomly chosen trials [74,75]. Zhang et al [127] and Köpcke et al [70] proposed case-based reasoning algorithms for free-text and structured patient data, respectively. These algorithms did not require the translation of eligibility criteria into rules, but tried to determine the unknown eligibility of new patients by comparing them with a set of patients with known eligibility status.

### Reasoning

#### Overview

Closely tied to the previously described CTRSS characteristics is the reasoning process itself (ie, the method to assess whether the available data for a patient suffices for the conditions set by the trial’s eligibility criteria). Almost all CTRSS “perform ‘pre-screening’ for clinical research staff” [115] instead of trying to determine the actual eligibility of a patient. They do not replace manual chart review, but act as a filter that limits the number of patients who require such by selecting the most likely candidates. The presentation of reasoning details, such as a probability of eligibility or missing patient characteristics together with the screening list, can facilitate the manual screening process even further.

The dominance of relational databases for the storage of patient data entails that most CTRSS employ database queries somewhere in the reasoning process. Consequentially, most CTRSS are based on an elaborate query or a set of subsequently executed queries per trial [3,45,64,126]. If the result set of potentially eligible patients is sufficiently accurate, no further processing is required.

Some authors demonstrated the feasibility of more exotic reasoning methods. A total of 4 CTRSS used Arden syntax to control the reasoning process [64,75,91,95]; 3 CTRSS employed an ontologic reasoner after transforming eligibility criteria and, in 2 cases, patient data into separate ontologies [35,72,96]. However, although technically interesting, the authors failed to convey the advantages of these algorithms when compared with the aforementioned simpler ones.

#### Dealing With Incomplete Data

Some CTRSS designers paid particular attention to missing patient data. Tu et al [119] developed 2 methods for dealing with this problem. In their qualitative method, each criterion was attributed 1 of 5 qualities according to a patient’s concrete data: patient meets the criterion, patient probably meets the criterion, no assertion possible, patient probably fails the criterion, and patient fails the criterion. Specific rules for each criterion derived one of these qualities from the patient’s data or assign default values. In their probabilistic method, a Bayesian belief network was manually constructed for each trial. The network represented variables as nodes and dependencies as links between nodes. All nodes and links were given probabilities based on legacy data or experts. If data for a variable were found, the variable was given a probability of 1 or zero; otherwise, the default probabilities were used. When all available data for a patient were retrieved, a probability for the patient’s eligibility could be calculated. This probabilistic approach was applied again later by Papaconstantinou et al [94] and Ash et al [31]. Bhanja et al [36] suggested that scalability as well as time and design complexities discouraged the use of probabilistic approaches.

#### Natural Language Processing

The wish to include unstructured (ie, free-text) data could also warrant the utilization of complex reasoning algorithms. Keyword searches were often employed when no structured data elements were available [29,41,73,93,101,106,110]. They could easily be added to complement queries of structured patient data [3,66,98,124]. Pakhomov [93] compared a keyword search with 2 other NLP methods: naive Bayes and perceptron. Naive Bayes yielded the best sensitivity (95% vs 86% and 71% for perceptron and keyword search, respectively) and perceptron offered the best specificity (65% vs 57% and 54% for naive Bayes and keyword search, respectively). Although performing worst of all methods, the advantage of using a simple keyword search lies in its easy implementation (no need for training data) and transparency. In a similar comparison, Zhang et al [127] found regular expressions outperformed a vector space method and latent semantic indexing to achieve accuracy similar to a specifically developed method called subtree match. However, they also proposed algorithms for automatic keyword and subtree generation, which could offer distinct potential for automation.

#### Sensitivity-Versus-Specificity Tradeoff

**Figure 3:**
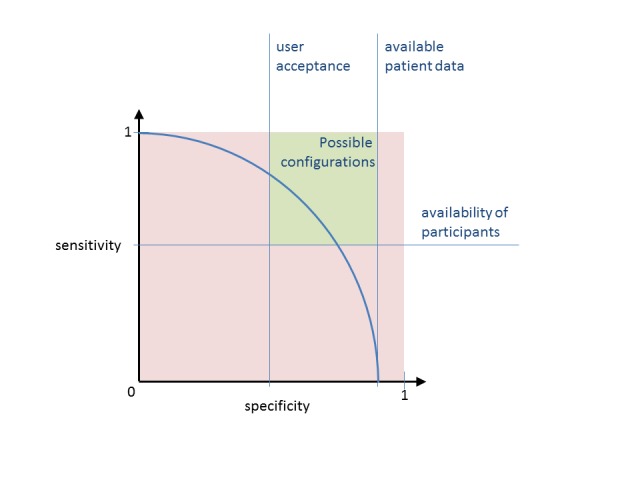
The sensitivity and specificity of patient proposals from most CTRSS depends on the configuration of the reasoning algorithm. The developer is free to favor specificity over sensitivity or vice versa, depending on conditions that are likely to be different for each trial. Frequently relevant conditions are user acceptance, the availability of patient data, and the availability of participants compared to the required number.

Independent from the chosen reasoning, the inclusiveness of each CTRSS is subject to the desires of its user. Ultimately, the setup of a CTRSS “requires sensitivity-versus-specificity tradeoffs” for each trial [119]. The upper limit to specificity might be determined by the fit between available patient data and eligibility criteria, whereas its lower limit is simply determined by what the user is willing to accept (Figure 3). The required level of sensitivity is limited by the availability of trial participants. Sensitivity should be chosen as low as possible to increase specificity and, thus, reduce recruitment workload. In practice, however, when the CTRSS is motivated by a lack of participants for a specific trial, maximum sensitivity is imperative and low specificity must be accepted [49].

### Outcome

#### Overview

All studies in this review shared the common goal to improve the recruitment process of clinical trials. However, calculating the performance of the CTRSS in terms of specificity and sensitivity alone is, at best, a secondary indicator for its effect. Direct comparison with the manual recruitment process with regard to its effects on one or more of the following 3 variables should be favored: (1) the pure number of trial participants (ie, the effectiveness of the recruitment process), (2) the cost to recruit a given number of patients in terms of money and/or time (ie, the efficiency of the recruitment process), and (3) the quality of the collective of trial participants (eg, measures for selection bias and dropouts). All reported system effects were weighted according to the scientific quality of the evaluation as (1) reliable quantitative measurement, (2) quantitative measurement with insufficient description of or flawed method, or (3) survey or estimation (corresponding to A-C in Multimedia Appendix 1, respectively).

#### Impact on Recruitment Effectiveness

We found 5 papers that reliably quantified differences in recruitment effectiveness between manual and CTRSS-supported recruitment. Embi et al [49] reported on a doubling of physician’s enrollment rate from 3 to 6 per month, which was attributed to a concurrent significant increase in the number of referring physicians from 5 to 42. The CTRSS presented by Cardozo et al [41] increased identification of eligible patients from 1 in 2 months to 6 in 2 months after physicians failed to generate pager notifications in time. Herasevich et al [63] doubled monthly enrollment rates from 37 in approximately 8.5 months to 68 in approximately 9 months in a time-critical setting. They attributed the effect to the change from imprecise clinical notes (manual process) to specific physiologic criteria (automated process) as the basis for eligibility evaluation. Beauharnais et al [34] also doubled recruitment, in this case from 11 patients in 63 days to 20 patients in 62 days. The effect seemed to correlate with an increase in screening efficiency that similarly doubled the number of screened patients. A comparatively minor increase in recruited patients of 14% from 306 to 348 in the same week was reported by Köpcke et al [69] who addressed pure oversight of otherwise well-organized manual recruiters. They also found 7% of the manually included patients did not fulfill the trial’s eligibility criteria.

Lane et al [71], Tu et al [119], and a research group from the University of South Florida [55,56,68,88] ran their respective CTRSS on legacy patient data and evaluated how many of those patients found potentially eligible by their system were actually enrolled in the past. These works only showed an upper limit of CTRSS effectiveness because it was unclear whether “physicians actually missed the matches, rather than having undocumented reasons for omitting them” [56]. Similarly, Weiner et al [122] described an increase in the number of eligibility alerts sent to the trial investigator. Again, these can only be an upper limit for the effect of the CTRSS on enrollment because the physician’s reasons for not alerting the investigator were unclear. It is possible that the physicians judged patients unfit for the trial for reasons beyond the criteria that were considered by the CTRSS or that the patients were unwilling to participate. Séroussi and Bouaud [108], Weng et al [124], and Treweek et al [118] compared the effectiveness of their CTRSS with conventional methods of recruitment by running them in parallel over the whole study period. However, the lack of enrollment numbers for a preceding phase without the CTRSS made it impossible to quantify the effect of the CTRSS. Finally, Ferranti et al [54] reported an increase in recruitment numbers by 53%. Although we found their methodology suitable, the authors failed to discuss reasons for a sharp increase in recruitment numbers 2 months before introduction of the CTRSS.

#### Impact on Recruitment Efficiency

We judged 4 papers to reliably quantify differences in the efficiency of a CTRSS and the manual recruitment process. Thompson et al [117] reduced the screening time required per eligible patient from 18 to 6 minutes (66%) in a 2-week evaluation of their CTRSS prototype. This reduction was achieved solely through a higher fraction of eligible patients among screened patients, whereas the individual screening time was actually higher for patients proposed by the CTRSS. Penberthy et al [98] verified this circumstance for 5 additional trials, achieving screening time reductions of 95%, 34%, 86%, and 34% in 4 trials and an increase of 31% in 1 trial. Again, time savings resulted from screening fewer noneligible patients, whereas individual screening time remained unchanged. Therefore, the benefit in efficiency was found to depend on the specificity of the CTRSS. Nkoy et al [3] decreased screening time from 2 to zero hours daily with no manual control of the patient list generated by their CTRSS. They translated these time savings into cost savings of US $1200 per month. Beauharnais et al [34] halved screening time from 4 to 2 hours daily, measuring manual and CTRSS-aided recruitment over 60 subsequent days, respectively. They concluded that “the use of an algorithm is most beneficial for studies with low enrollment rates because of the long duration of the accrual period.”

Following a proposition by Ohno-Machado et al [90], the aforementioned research group from the University of South Florida [55,56,68,88] presented a unique approach to increase screening efficiency. Through ordering of the necessary clinical tests for eligibility determination in such a way that cheap but decisive tests were done first, they expected a reduction of costs by 50%. The cost of each test and the number of clinical trials and eligibility criteria that required a test’s results were included in the calculation. Unfortunately, the evaluation of the methodology was based on retrospective data and it remained unclear how the cost for tests without reordering were calculated. Seyfried et al [110] reported decreased screening time, but used the same dataset with the same test physicians for both manual and CTRSS-aided screenings (50 patients, 1-week interval). Furthermore, the CTRSS appeared to be trained with the same dataset on which it was tested later. Thadani et al [115] and Schmickl et al [106] did not directly measure screening time decreases, but stated that they could imagine screening only patients proposed by their respective CTRSS to be sufficient, reducing the patient pool by 81% and 76%, respectively. Obviously, such a strategy would require the CTRSS to feature a sufficiently high sensitivity.

#### Impact on Recruitment Quality

Only Rollman et al [104] compared the characteristics of patient sets after manual and CTRSS-aided recruitment. To this end, they observed 2 subsequent trials with similar eligibility criteria, the same recruitment period of 22 months and the same 4 recruiting primary care physicians. They found that usage of the CTRSS significantly increased the proportion of male nonwhite patients, as well as the fraction of patients with more severe disease grades.

## Discussion

### Principal Findings

There are some CTRSS setups that reappear on a regular basis. Firstly, for the retrospective identification of trial participants based on existing clinical data, database queries are designed and executed once or on a regular basis. They create a list of potentially eligible patients that is printed on paper or otherwise delivered to the researcher. Secondly, for trials with short windows of opportunity for recruitment, a key event in the EHR or another health IT component is constantly monitored. Its occurrence causes a more comprehensive eligibility test for the concerned patient and is communicated to the researcher via pager. Thirdly, if no patient data exist yet, it is entered directly into the CTRSS, which assesses and communicates the patient’s eligibility directly after completion of data entry. Although the treating physician was required to act for the patient in older systems, it is now becoming increasingly popular to offer this possibility directly to the patient via dedicated websites. Our review confirms the findings of Weng et al [129] who also gave names to these CTRSS types: (1) mass screening decision support, (2) EHR-based recruitment alerts, and (3) computerized research protocol systems and Web-based patient-enabling systems (depending on the user).

The setup of a specific CTRSS is rarely chosen on a theoretical background (ie, after an evaluation of different options for triggering the system and communicating the results). Instead, the setup is dictated mostly by the existing clinical environment, available IT tools, and the needs of a specific trial or group of researchers. Because CTRSS are a subset of clinical decision support systems (CDSS), it will generally be possible to configure existing CDSS such that they assume CTRSS functionalities (eg, [50,64,86]).

### Limitations of the Review

Our review is limited in that the collection of publications and extraction of information from these publications was done by only 1 author. We reduced the impact of this approach by refraining from any interpretation of the given information in this step. Nevertheless, we cannot preclude mistakes, especially when stating that no or unclear information on a certain CTRSS characteristic was found in an article. Furthermore, all unreferenced statements made in this review reflect only the opinion of the 2 authors and are subject to discussion by the research community.

### Comparison With Previous Review

Our review of 101 CTRSS publications offers the most comprehensive and up-to-date overview on CTRSS. Compared to the previous review paper by Cuggia et al from 2011 [17], which analyzed 28 CTRSS from articles published before October 2009, we identified an increase of publications in the subsequent years. These more recent publications present more data on the impact of CTRSS on the recruitment process, which we discuss subsequently. Of the 7 tendencies in CTRSS research formulated by Cuggia et al, all but the exclusive reliance on structured data appear to continue. We found many CTRSS that include unstructured data as a data source, although many of them are limited to keyword searches. There are 3 additional lessons we believe can be learned from the existing research, which are described subsequently.

Lack of standards is not limited to the terminologies of the patient data source, but also applies to the computational representation of eligibility criteria. Although researchers have proposed independent languages to encode the free-text criteria of a trial’s protocol (eg, ERGO [130], EliXR [131]), most CTRSS bind the representation of eligibility criteria in 2 ways to the specifics of their environment: (1) to the terminology of the patient data source and (2) to the chosen reasoning method. We believe independent and exchangeable eligibility criteria to be desirable because multisite trials have become the norm. However, judging from the experience so far, readily encoded criteria will need to be the norm in trial protocols before they will be adopted by CTRSS designers. Tools to help translate the criteria into SQL statements could speed up the adoption process.

The choice of the reasoning method should consider its pervasiveness (ie, how easily third parties interested in its deployment can learn to install and administrate it). Considering this, no other method seems to be as suitable for CTRSS as SQL queries on relational databases. Queries can make use of existing data from the EHR, a data warehouse (DWH), or a registry and their administrators are likely to be experienced creators and users of such queries. Resistance to adopt and maintain an additional query-based system is likely to be small compared to CTRSS that require additional training in one of the less widespread technologies, such as probabilistic methods or Arden syntax. Although complex reasoning methods have been shown to achieve high accuracy, it is unclear whether they lead to an increased CTRSS impact compared to queries.

Using patient care data promises efficiency and effectiveness gains for a CTRSS. But, because it is collected for other purposes, it also introduces new challenges [131]. It is imperfect from the viewpoint of eligibility assessments because it lacks uniformity (the same information can be documented differently for 2 patients), timeliness (information might be documented too late), and completeness (information might be missing for some or all patients). Uniformity and completeness problems can lead to severe selection bias and increase the cost of eligibility rule creation. For example, low uniformity necessitates an analysis of documentation habits; low completeness might enforce the use of proxy data [78] or estimates [90]. Timeliness must be ensured by the documentation process, which might resist change. Untimely data will severely limit the possibility to support a trial, especially in outpatient settings [46]. Thus, unfit data can constitute a major limitation to CTRSS impact.

### First Conclusions on Clinical Trial Recruitment Support Systems Impact

We suggested that the introduction of a CTRSS can be motivated by 3 expectations: (1) an increase in the number of participants for a given clinical trial or a set of trials, (2) a reduction of trial costs through decreased screening costs, and (3) the guarantee to select a representative set of patients (ie, the reduction of selection bias). Many authors do not elaborate on the shortcomings of the manual recruitment process that led to the development of their CTRSS.

Whether a CTRSS is able to increase the number of participants for a trial depends little on its setup, but rather on the deficits of the manual recruitment process it is set to replace. To begin with, an untapped group of potential participants (ie, a gap between those patients who are eligible and those who are asked to participate) needs to exist. This gap originates from some patients not being screened at all or from communication problems between the different actors of the recruitment process. Thus, a CTRSS can close this gap if it can ensure that every patient is screened and that the necessary information on the patient and the trial is available in time.

Often, a CTRSS is expected to close the gap between estimated and realized participant numbers or that between eligible and recruited patients or even the gap between needed and available patients. These expectations are likely to be disappointed. They disregard that many causes for insufficient recruitment are out of the scope of a CTRSS or simply cannot be addressed by an IT intervention. The most important is a willingness of the patient to participate and motivation of physicians to participate in recruitment. The analysis of the existing recruitment process and its weaknesses should, therefore, be part of every CTRSS design process. Weng et al [123,124] give examples on how to do this. They characterize patient eligibility status in different categories, such as potentially eligible, approachable, consentable, eligible, and ultimately enrolled. By comparing the ratio of patients in each category, such taxonomy can be used to identify the weak spots in recruitment that need to be addressed by the CTRSS.

Although the effectiveness of a CTRSS is determined by its setting, improvements in screening efficiency might be more generally achievable. Many successes to reduce screening time are based on using existing data to reliably exclude patients from the screening list (ie, the CTRSS generates no or few false negatives). In this way, the CTRSS can be used to reduce the number of patients that must be screened manually. Under the reasonable assumption that documented data are correct, but not all patient characteristics are documented, we believe CTRSS should focus on the exclusion criteria of a clinical trial to maximize efficiency gains. No final eligibility decision should be based on the trial’s inclusion criteria because this can reduce the sensitivity of the CTRSS and motivate the screeners to use other screening methods in parallel. To realize efficiency gains, the CTRSS must completely replace the former screening process. This also means that the aim to increase recruitment efficiency is opposed to the other 2 potential aims of a CTRSS which profit from running multiple screening methods in parallel.

The potential benefit of a CTRSS on the composition of a trial’s participants has been insufficiently explored so far. Because patient demographics should be easily obtainable for all experiments comparing manual and CTRSS-aided recruitment, we suggest including them in future publications.

### Future Directions

We found most articles describe the characteristics and operating principles of their CTRSS reasonably well, but all lacked in some regard. Intermediary criteria representation, terminologies of the patient data, and an evaluation of the system’s effects were often missing. Many authors present prototypes of their CTRSS directly after finishing its design and fail to report on its outcome and usage. We encourage more follow-up publications on the experiences with existing CTRSS such as those by Embi et al [51], Embi and Leonard [52], and Dugas et al [47]. To strengthen the comprehensibility and usefulness of future reports, we propose a list of essential elements that should be included (Textbox 1).

In their review of patient cohort identification systems in general, Shivade et al [21] found a “growing trend in the areas of machine learning and data mining” and believe these necessary to develop generalizable solutions. For CTRSS in particular, this trend has not yet manifested in the literature. Only Zhang et al [127] and Köpcke et al [70] report on experiments to exploit these techniques for recruitment purposes, but both are still in a prototype stage. Machine learning promises more independence from the individual representation of patient data in a hospital and better portability. Still more data are needed to assess advantages and disadvantages and to explore hybrid solutions.

**Essential elements to be included in future CTRSS studies.**

Clinical Context

1. Number of trials that the CTRSS has been evaluated for
2. Length of time the CTRSS has been in use
3. Brief description and number of sites that use the CTRSS

Input

1. Representation format of eligibility criteria in the CTRSS
2. Comparison of original and computable eligibility criteria for an exemplary trial
3. Summary of how well the eligibility criteria could be translated for all other trials (if any)
4. Details on the translation process
5. Representation of patient data in the CTRSS
6. Details on the patient data source (eg, purpose, terminologies)

Working Principle

1. What triggers an eligibility assessment?
2. How are eligibility criteria and patient data compared?
3. How is the result of the assessment communicated, when is it communicated, and to whom?

Outcome

1. Recruitment process before introduction of CTRSS including perceived problems
2. Recruitment process following introduction of CTRSS
3. Patient numbers and time spent for each step

Current publications in the area of CTRSS are still too focused on—and sometimes limited to—technical aspects of system setup and the accuracy of its eligibility assessment. After review of most of the existing literature, we believe that the impact of a CTRSS on a given recruitment process is determined more by the context of the CTRSS (ie, the available patient data, its integration in trial, and clinical workflows and its attraction to users). Therefore, what is needed are research projects to evaluate how a CTRSS can be embedded in different recruitment workflows, the characteristics of trials that profit from CTRSS, different designs for user interaction, and the outcomes of CTRSS in relation to these parameters.

### Conclusions

We further found that differences in the setup of CTRSS are because of existing infrastructure and particularities of the recruitment process, particularly the target user of the CTRSS (eg, treating physician, study nurse) and the prior recruitment problem (eg, failure to identify, failure to communicate). Yet, there are still many questions open in defining when and how CTRSS can best improve recruitment processes in clinical trials. Based on the questions that remained open in our analysis of many of the 101 articles, we propose an item list that should be considered for future publications on CTRSS design, implementation, and evaluation. This shall ensure that CTRSS setup and background, their integration in research processes, and their outcome results are sufficiently described to allow researchers to better learn from other´s experiences.

## Multimedia Appendix 1

Table of publications included in qualitative analysis together with major CTRSS characteristics.

## Abbreviations

CDE: common data elements
CTRSS: clinical trial recruitment support systems
EHR: electronic health record
ERGO: Eligibility Rule Grammar and Ontology
HIS: hospital information system
ICD: International Classification of Diseases
LOINC: Logical Observation Identifiers Names and Codes
MED: Medical Entities Dictionary
MLM: Medical Logic Module
NCI: National Cancer Institute
NLP: natural language processing
SNOMED CT: Systematized Nomenclature of Medicine Clinical Terms
SQL: Structured Query Language
UMLS: Unified Medical Language System
